# Contrast-enhanced ultrasound and microwave ablation of Epstein–Barr virus associated smooth muscle tumors in a patient with AIDS: a case report

**DOI:** 10.3389/fmed.2024.1373786

**Published:** 2024-09-30

**Authors:** Yuanping Yang, Xiumei Bai, Yuquan Wu, Hong Yang, Yun He

**Affiliations:** Department of Medical Ultrasound, First Affiliated Hospital of Guangxi Medical University, Nanning, China

**Keywords:** microwave ablation, contrast-enhanced ultrasound, HIV, EBV, smooth muscle tumor

## Abstract

This case report presents a rare case regarding the contrast-enhanced ultrasound (CEUS) features and minimally invasive treatment of Epstein–Barr virus associated smooth muscle tumors (EBV-SMT) in a patient with human immunodeficiency virus infection (HIV). Currently, there are few studies involving hepatic EBV-SMT. According to WHO guidelines, the malignant potential of the disease is uncertain. The features of CEUS suggest that these hepatic lesions tend to be malignant and are often misdiagnosed as other malignant neoplasms. Presently, hepatic resection is the first choice for treating hepatic EBV-SMT. However, immunocompromised patients may benefit more from minimally invasive microwave ablation therapy (MWA). Since there is no standard diagnosis and therapy are available at present, our findings in this case may contribute to promoting standardized diagnosis and treatment of EBV-SMT.

## 1 Introduction

Epstein–Barr virus (EBV) has the effect on promoting smooth muscle proliferation and inducing EBV-associated smooth muscle tumors (EBV-SMT). EBV-SMT is a rare mesenchymal tumor that occurs primarily in immunocompromised individuals (1–3). There are three types of EBV-SMT: human immunodeficiency virus-associated smooth muscle tumor (HIV-SMT), congenital immunodeficiency disorder-associated smooth muscle tumor (CI-SMT) and followed by post-transplantation smooth muscle tumor (PT-SMT) (4–6). Currently, most EBV-SMT cases are still known through case reports. Evidence and guidelines for the diagnosis and treatment of EBV-SMT are still lacking.

Thus, we reported an interesting case of hepatic EBV-SMT in an adult patient with HIV infection. This case’s contrast-enhanced ultrasound (CEUS) characteristics were also presented in detail. Additionally, we have reported for the first time the application of microwave ablation (MWA) in treating hepatic EBV-SMT and achieved good therapeutic effects.

## 2 Case report

A 23-year-old male had a persistent fever for 10 days with a peak temperature of 39.6°C, and was accompanied by abdominal pain symptoms. The patient has a personal history of smoking and drinking. After admission to our hospital, HIV, hepatitis B and liver fluke infection workup was positive for the patient. The viral DNA levels for Hepatitis B were 1.13×10^7^ IU/ml. Additionally, CD4(+) T cell counts decrease, and CD8(+) T cell counts increase, and the ratio of the former to the latter decreases (CD4(+)/CD8(+) = 0.02, <0.5). All other laboratory tests, including routine blood, liver function, kidney function, coagulation, and tumor markers, et al., were within normal limits. Meanwhile, the remainder of his vitals were normal, and his physical examination was unrevealing.

**Figure 1:**
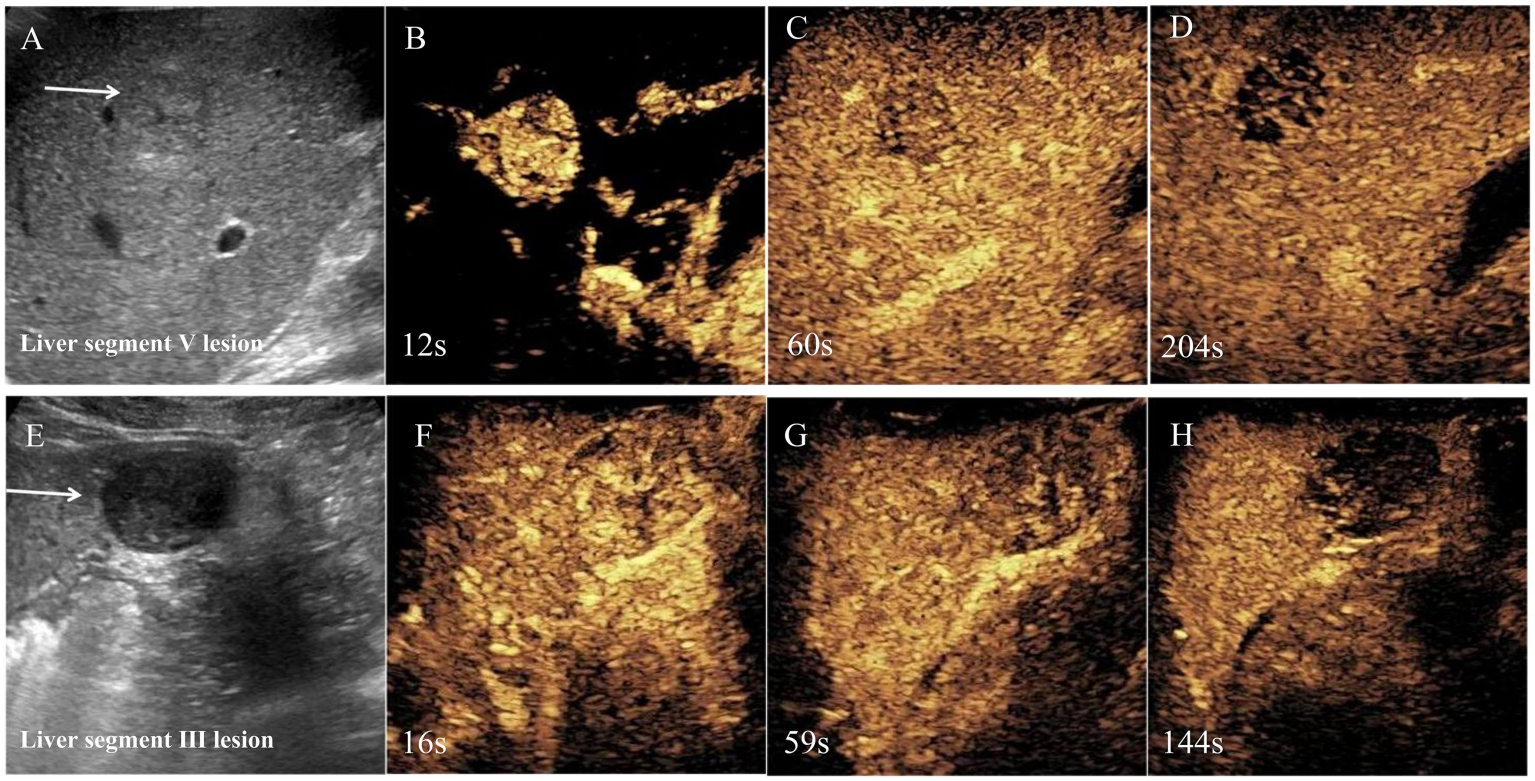
Regular ultrasound and contrast-enhanced ultrasound of segments V and III lesions. Regular ultrasound was shown there were two hypoechoic lesions in segments V and segments III, respectively **(A,E)**. Contrast-enhanced ultrasound was performed and the lesions displayed obvious heterogeneous enhancement in the arterial phase **(B,F)**. The portal venous phase showed hypoenhancement **(C,G)**, and the late phase showed marked washout **(D,H)**. Based on the enhanced ultrasound imaging features, malignant tumor was considered.

**Figure 2:**
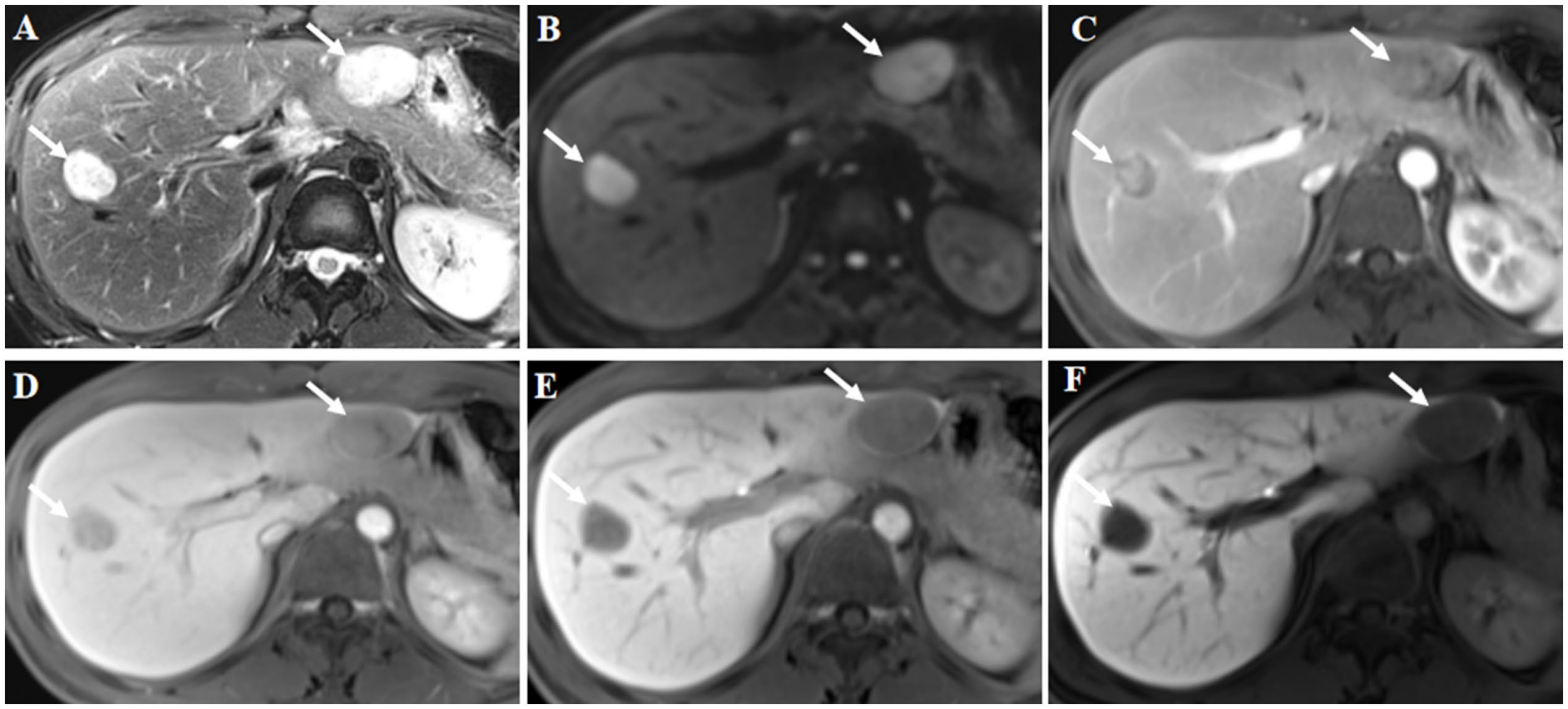
Gd-EOB-DTPA magnetic resonance imaging (MRI) findings. The lesions were located in liver segments V and III, and showed high signal intensity on axial T2-weighted imaging (**A**, arrow). Diffusion weighted image showed diffusion restriction (**B**, arrow). Contrast-enhanced MRI showed inhomogeneous enhancement of the lesions at arterial phase (**C**, arrow). The signal intensity of the lesions was lower at the portal phase (**D**, arrow) and delayed phase (**E**, arrow) compared with the surrounding liver parenchyma. Hypointensity was noted in the hepatobiliary phase at 30 min post-injection (**F**, arrow). Hepatocellular carcinoma was considered based on the findings of the magnetic resonance imaging.

Abdominal ultrasound examination of the liver indicated that there were two focal liver lesions at the segments V and III (Figures 1A,E). Subsequently, the liver CEUS was performed to further characterize the liver lesions (Figures 1B–D,F–H). The inspection results demonstrated that CEUS in both two lesions revealed homogenous hyperenhancement in the early arterial phase, followed by early washout in the portal venous phase (Figures 1B,C,F,G). Overall, both of the lesions showed a typical malignancy appearance and were diagnosed as malignant tumors. Additionally, further workup with Gd-EOB-DTPA magnetic resonance imaging (MRI) demonstrated the focal liver lesions at the segments V and III, leading to the same diagnosis (Figure 2).

A multidisciplinary team conducted consultations on the patient’s condition. Ultimately, owing to the patient’s immunocompromised status, treatment was carried out in a staged procedure. Since segment III lesions are close to the gastrointestinal tract, MWA poses a risk. Given this situation, it was decided to first utilize ultrasound-guided percutaneous biopsy, followed by MWA for the lesion at segment V and hepatic resection for the lesion at segment III after 2 weeks.

**Figure 3:**
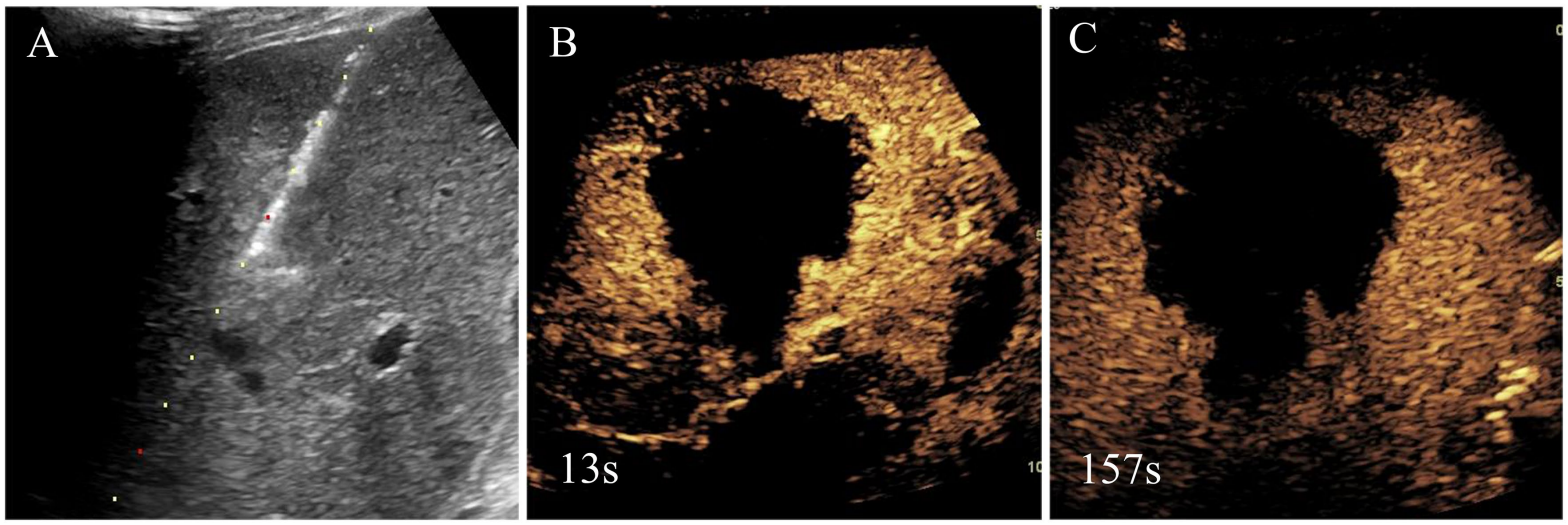
Needle biopsy, microwave ablation treatment and immediate contrast-enhanced ultrasound after microwave ablation of liver segment V lesion. Based on the clinical findings, the initial diagnosis by the multidisciplinary team was malignant tumor, and, thus, liver segment V lesion microwave ablation was performed **(A)**. Immediate contrast-enhanced ultrasound after microwave ablation was shown there was no enhancement in lesion and achieved complete ablation **(B,C)**.

**Figure 4:**
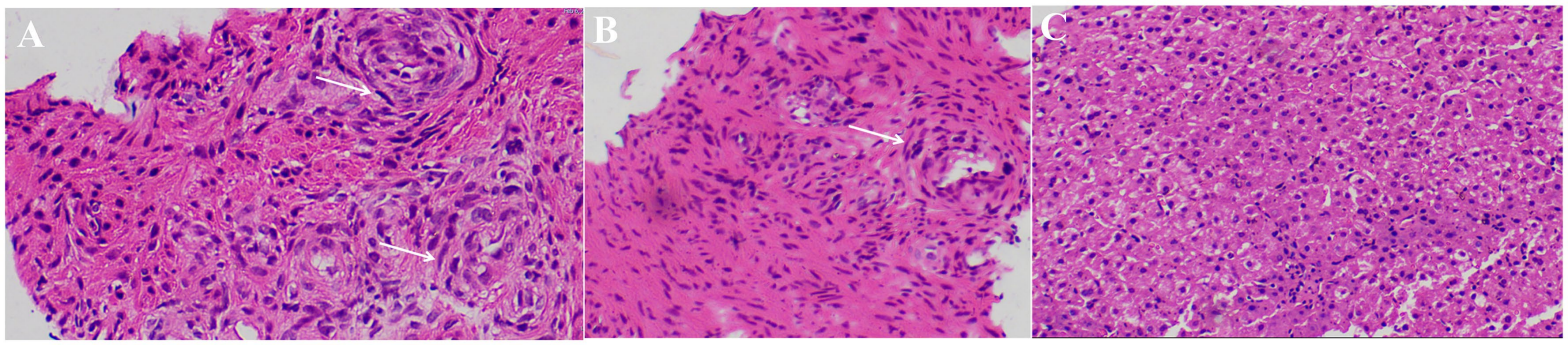
Pathological results of liver segment V lesion. Hepatic lobular structures can be seen **(A–C)**, and a few spindle cell nests was around it (white arrows).

Ultimately, the patient underwent MWA treatment for segment V lesion (Figure 3). Subsequently, the histological result suggested the tumor was composed of spindle cells (Figure 4). Smooth muscle tumor was diagnosed with immunohistochemistry, but further tests needed to be conducted to differentiate perivascular epithelioid cell tumors. Additionally, *in situ* hybridization indicated positive for EBV-encoded RNAs (EBERs) and showed evidence of EBV infection. Finally, the patient was diagnosed with EBV-SMT based on pathological results.

## 3 Discussion

Previous reports mainly focused on the pathogenesis and treatment of EBV-SMT, but there is little literature on the imaging characteristics of EBV-SMT. Most previous literature suggests that EBV-SMT does not have specific imaging features, and liver lesions are often multiple (6, 7). In this case, the diagnosis of EBV-SMT is easily confused with other malignant tumors. Here, we report an EBV-SMT case with HIV infection. According to the previous study, in patients with HIV-related EBV-SMT, hepatic lesions are more common in children and less in adults (7). Besides, the central nervous system, gastrointestinal tract, liver, lungs, skin, larynx, pharynx, and adrenal glands are the most commonly affected sites in HIV-related EBV-SMT cases (5, 8–10). This case report will provide some reference for the diagnosis and treatment of EBV-SMT.

To the best of our knowledge, this is the first report to describe the characteristics of two-dimensional (2-D) ultrasound and CEUS about hepatic EBV-SMT. Specifically, liver lesions exhibited hypoechoic masses, well-defined, and regular morphology features on 2-D ultrasound. In CEUS, arterial phase lesions indicated high enhancement, followed by low enhancement in portal venous phase and delayed phase. Thus, EBV-SMT can be easily confused with other malignant lesions, such as liver cancer.

So far, there is no unified treatment for EBV-SMT. The means of treatment include surgical resection, chemotherapy, radiotherapy, and highly active antiretroviral therapy, with surgical resection remaining the dominant treatment (6, 11, 12). Since EBV-SMT was first reported in the 1990s, the number of HIV-related EBV-SMT cases have increased (11, 13). Patients with HIV-related EBV-SMT have a worse prognosis compared with other types, which may be related to complications of HIV-related infection (14). Here, we reported for the first time that MWA may serve as a potentially superior alternative for patients with HIV-related EBV-SMT who cannot tolerate surgery. In this case, the patient underwent MWA and was actively treated with anti-infective therapy. The temperature of the patient returned to normal. Based on the experience of this case, we believe that MWA is also an effective treatment method for HIV-related EBV-SMT patients who are not suitable for surgery due to poor underlying conditions. Of course, antiviral therapy for patients is still crucial.

Overall, we firstly report a case of MWA treatment for EBV-SMT patients with HIV. Although the malignant potential of the disease is uncertain, the CEUS features of lesions indicate a tendency towards malignancy, presenting challenges in disease diagnosis. Given the comprehensive constellation of findings in this case, it may promote standardized diagnosis and treatment of this rare disease.

## Data Availability

The original contributions presented in the study are included in the article/supplementary material, further inquiries can be directed to the corresponding authors.
